# Retraction: Research on crime motivation identification and quantitative analysis methods based on EEG signals

**DOI:** 10.3389/fpsyg.2026.1840793

**Published:** 2026-04-01

**Authors:** Frontiers Editorial Office

The journal retracts the 18 March 2025 article cited above.

Following publication, concerns were raised regarding the validity of the data in the article. The authors failed to provide the raw data or a satisfactory explanation during the investigation, which was conducted in accordance with Frontiers' policies. Given the concerns, and the lack of raw data, the editors no longer have confidence in the findings presented in the article.

This retraction was approved by the Chief Editors of Frontiers in Psychology and the Chief Executive Editor of Frontiers. The authors received a communication regarding the retraction and had a chance to respond. This communication has been recorded by the publisher.

Frontiers would like to thank the concerned reader who contacted us regarding the published article.

